# Impact of Modified 2013 ASCO/CAP Guidelines on HER2 Testing in Breast Cancer. One Year Experience

**DOI:** 10.1371/journal.pone.0140652

**Published:** 2015-10-16

**Authors:** Zsuzsanna Varga, Aurelia Noske

**Affiliations:** Institute of Surgical Pathology, University Hospital Zurich, Zurich, Switzerland; University Medical Centre Utrecht, NETHERLANDS

## Abstract

**Introduction:**

The latest guidelines of the American Society of Clinical Oncology/College of American Pathologists (ASCO/CAP) to test Human Epidermal Growth Factor Receptor 2 (HER2) in breast cancer after being revised in 2008 underwent a second modification in October 2013. The modification includes changes in cut-offs: 10% strong membranous staining for score 3+ on immunohistochemistry (IHC) (previously 30%) and using the ratio of >2 or absolute gene-copy-number (6 or more) alone or in combination with each other by in-situ-hybridisation technology (previously >2.2 and average copy-number of 6 or more). Hereby we addressed the question, which impact the modified cut-offs had on overall HER2-positivity in a single institution.

**Methods:**

We prospectively analysed 617 consecutive diagnostic breast-cancer cases which underwent double HER2 testing by immunohistochemistry and fluorescent in-situ hybridisation (FISH), using the modified 2013 ASCO/CAP-guidelines for one year (October 2013–October 2014). Results were compared with HER2-test results on 1,528 consecutive diagnostic breast-cancer cases from two previous years (2011–2012), using the 2008 ASCO/CAP guidelines, also tested with IHC and FISH in each case.

**Results:**

Between October 2013 and October 2014, overall HER2-positivity was 15.8% (98 of 617 cases were either IHC 3+ or FISH amplified). 79 of 617 cases (13%) were IHC 3+, 96 of 617 cases (15.5%) were FISH amplified. Equivocal cases were seen in 25 of 617 cases (4.1%). 22 of 25 equivocal cases (88%) in 2013–2014 were IHC 1+ or 2+. In 13 equivocal cases, there was a repeated IHC/FISH testing: 2 of 13 cases (15%) became FISH amplified, 1 of 13 cases (7.5%) became IHC 3+. In 2011–2012, overall HER2-positivity (IHC/FISH) was 13.8% (211 of 1,528 cases). 185 of 1,528 cases (12%) were 3+ on IHC, 181 of 1,522 cases (12%) were amplified by FISH. Six of 1,528 cases were equivocal by FISH, and interpreted as non-amplified (0.3%).

**Conclusions:**

Applying the modified ASCO/CAP guidelines from 2013 resulted in an increase (2%) in overall HER2-positivity rate compared to overall-HER2-positivity rate using the 2008 ASCO/CAP guidelines. The increased positivity rate was mainly due to more FISH-positive cases (3.5% more than until 2013). The high rate of equivocal cases (4.1%) did not contribute to increase in overall HER2-positivity, but resulted in delay in definitive HER2-status.

## Introduction

The assessment of HER2 status in breast cancer is a part of routine tests on primary and / or on recurring lesions [1–3]. HER2 assays are increasingly carried out on preoperative core needle or vacuum assisted breast biopsies due to preoperative therapy planning particularly in terms of neoadjuvant chemotherapy options. Thus surgical breast specimens serve as additional options for HER2 assays as well. International guidelines for scoring of immunohistohemical and in situ hybridisation signals are available and widely used in HER2 testing in breast cancer [1, 3–5] Practising pathologists, being involved in breast pathology diagnostic service, need special training and long term expertise in order to achieve reproducible and standardized HER2 test results using the international guidelines [2, 6, 7]. Exact rate of HER2 positive breast cancer within a given institution or in a predefined timeframe or geographic area, are estimated upon the positivity rate, reported in the pathology reports. Modifying scoring criteria can consequently result in increase or decrease of reported HER2 positivity [6]. The first adjustment of the original Federal Drug Administration (FDA) guidelines and their replacement by the ASCO/CAP guidelines in 2008 resulted in a decrease of overall HER2 positivity rate from 17–20% to 13–14% [3, 6]. However, additionally to cut-off modification in the guidelines, the introduction of mammography screening and the detection of smaller tumors may have also contributed to the decrease in HER2 positivity rate [6]. The ASCO/CAP HER2 testing guidelines underwent a revision some years ago, and the newly published recommendation in October 2013 resulted in modification of HER2 scoring criteria again [4]. Basically, the cut-offs were downgraded and the definition of polysomic cases and the significance of the HER2/CEP17 ratio were newly defined.

In this study we compared overall HER2 positivity rate prior to the introduction of the modified ASCO/CAP guidelines from October 2013 with the ones using the new definitions from this time.

We prospectively analysed consecutive HER2 diagnostic cases from the period of 2011–2012 (n = 1528) and compared with HER2 test results between October 2013 and October 2014 (n = 617). All breast cancer cases underwent double HER2 testing by immunohistochemistry and fluorescence in situ hybridisation according to the institutional guidelines as published earlier [6]. We addressed the question how the modification of the guidelines and the cut-offs influenced the HER2 positivity rate and what impact resulted after this modification on diagnostic HER2 testing in breast cancer.

## Material and Methods

### Patient’s cohort

Consecutive patients with the diagnosis of invasive breast carcinoma undergoing HER2 testing at the Institute of Surgical Pathology, University Hospital Zurich, Zurich, Switzerland were analysed. Two periods were evaluated, 2011–2012 (two years) and from October 2013 to October 2014 (one year). Data on HER2 status from 2011–2012 were partially included in a previous paper, cases with polysomy or equivocal HER2 status were not included in this previous study [6]. In the period of 2011–2012, there were 1528 patients analysed, in the period of October 2013-October 2014, there were 617 patients, who underwent HER2 testing. The study was a prospective data analysis of an existing data bank without any additional experiment on human tissue. This study is a part of a previously approved project by the Ethical Committee of Canton Zurich (KEK-2012-553) and was conducted in a completely anonymized way. Informed consent was not required for the study due to the anonymity of the data used.

### Laboratory data

During the whole period (2011–2014), the same laboratory procedure for IHC and FISH was applied as published earlier [6].

### Diagnostic criteria 2011–2012

Protein expression and gene copy number score were scored using the time current ASCO/CAP guidelines as published earlier [3, 6].

#### HER2 immunohistochemistry

Score 0: no staining.

Score 1+: weak and incomplete membrane staining in less than 10% of the invasive tumor cells.

Score 2+: weak complete staining of the membrane in more than 10% of invasive cancer cells.

Score 3+: strong complete homogenous membrane staining in more than 30% of the invasive tumor cells.

#### HER2 FISH

HER2 signals were scored as follows: the number of signal copies and the ratios (HER2/CEP17) were calculated: gene copies (> 6) or cluster formations (small clusters ~ 6 copies, larger clusters ~ 12 copies) were counted. A ratio > 2.2 was set as amplified status; a ratio < 1.8 was negative, and a ratio of 1.8–2.2 was referred to as equivocal (according the 2008 guidelines) and using ratio > 2.2 and > 6 HER2 gene copies (in > 10% of the tumor cells) for a positive status (according to 2008 guidelines). If ratio was <2.2, the gene copy number was not considered alone as amplified based on the definitions in the recommendations in Tables 2 and 4 in the ASCO/CAP 2008 guidelines for dual FISH probes [3]

### Diagnostic criteria 2013–2014

Protein expression and gene copy number score were scored using the latest ASCO/CAP guidelines published in October 2013 [4].

#### HER2 immunohistochemistry

Score 0: no stain or faint incomplete membrane stain in not more than 10% within the cells. Score 1+: weak and incomplete membrane staining in more than 10% of the tumor surface. Score 2+: Complete intense membrane staining in not more than 10% of the invasive tumor cells or weak/moderate heterogeneous incomplete staining in more than 10% of the invasive tumor cells.

Score 3+: strong complete homogenous membrane staining in more than 10% of the invasive tumor cells.

#### HER2 FISH

HER2 gene copy signals were scored as follows:

Positive status was defined either as an average HER2 gene copy numbers of 6 at cases with a HER2/CEP17 ratio of less than 2 or a HER2/CEP17 ratio of 2 or more independently of the average gene copy number.

Negative status was defined as average gene copy number of less than 4 with a HER2/CEP17 ratio of less than 2.

Equivocal cases were defined as average gene copy number of at least 4 and less than 6 with a HER2/CEP17 ratio of less than 2.

## Results

**Fig 1:**
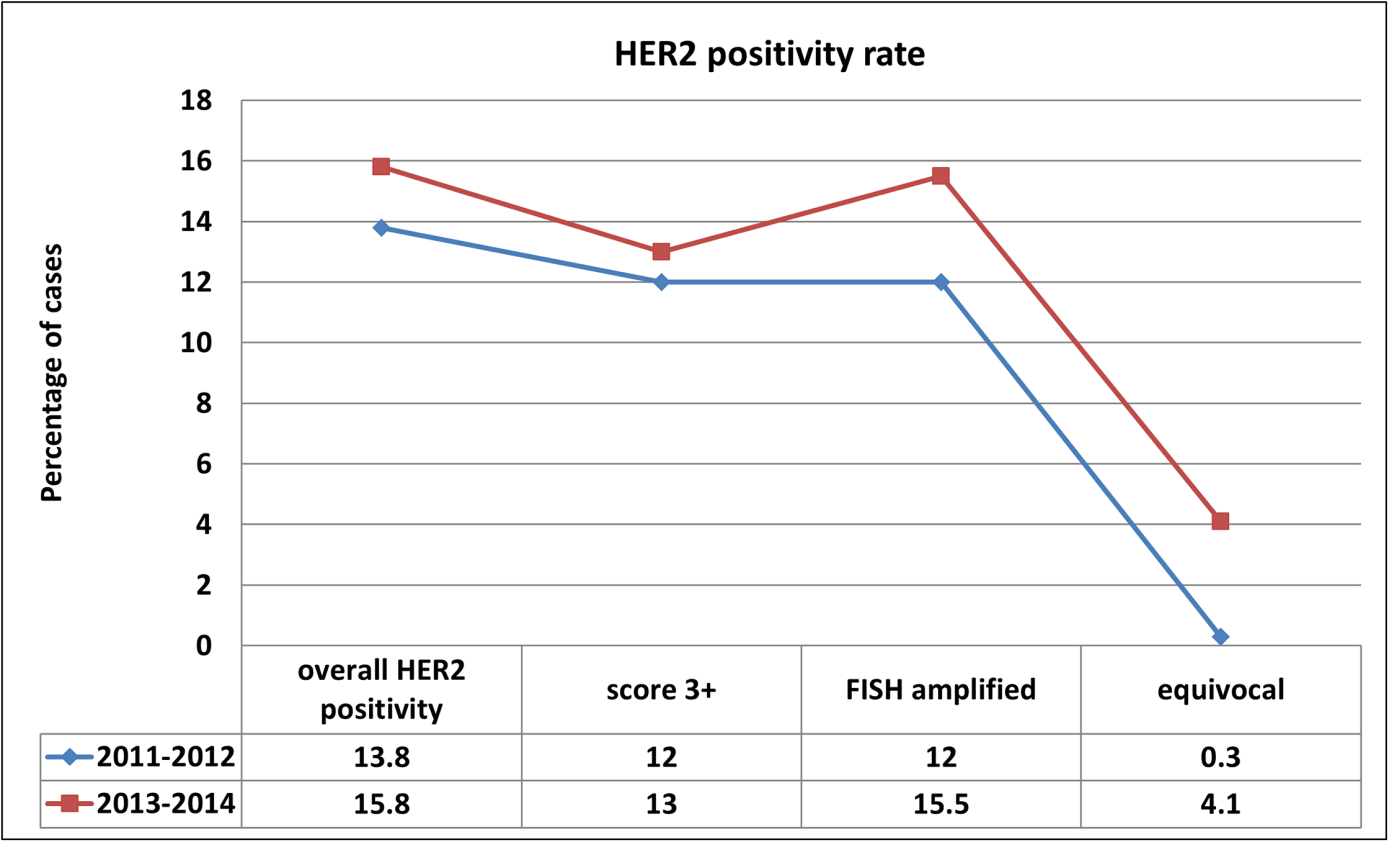
Diagrammatic representation of HER2 positivity rate in percentage in the periods of 2011–2012 (two years) and from October 2013 to October 2014 (12 months).

Comparison of the overall HER2 positivity rate in two periods with at the time current guidelines (ASCO/CAP 2008 and 2013) [3, 4] are shown in **Fig 1.**

### 2011–2012

Overall HER2 positivity (IHC score 3+ or amplified by FISH) was 13.8% (211 of 1528 cases). 185 of 1528 cases (12%) were 3+ on IHC, 181 of 1528 cases (12%) were amplified by FISH.

Six of 1528 cases were equivocal on FISH (0.3%). The one case with IHC score 3+ was considered as HER2 positive. The other five cases underwent repeated IHC/FISH testing on the core biopsy or on a different tumor block, and none of them became amplified or IHC positive.

Data are shown in **Table 1.**

**Table 1:** Concordance of test results in routine diagnostic HER2 assays in breast cancer. Immunohistochemistry (IHC) and FISH in 2011 and 2012.

	IHC score 0	IHC score 1+	IHC score 2+	IHC score 3+
FISH amplified	3 (<1%)	3 (<0.5%)	20 (5%)	155 (84%)
FISH non-amplified	361 (>99%)	567 (>99%)	384 (94%)	29 (15.5%)
FISH equivocal	-	1 (<0.5%)	4 (<1%)	1 (0.5%)
FISH monosomy	-	-	-	-
N = 1528	364 (100%)	571 (100%)	408 (100%)	185 (100%)

### 2013–2014

Overall HER2 positivity (IHC score 3+ or amplified by FISH) was 15.8% (98 of 617 cases were either 3+ on IHC or amplified by FISH. 79 of 617 cases (13%) were 3+ on IHC, 96 of 617 cases (15.5%) were amplified by FISH.

Equivocal cases by FISH were seen in 25 of 617 cases (4.1%). 22 of 25 equivocal cases (88%) were either scores 1+ or 2+ on IHC. In 13 equivocal cases, there was a repeated IHC/FISH testing: 2 of 13 cases (15%) became amplified by FISH, 1 of 13 cases (7.5%) was 3+ on IHC. The other 10 cases remained negative or equivocal by FISH and score 1+ or score 2+ by IHC. Altogether 3 of 13 (23%) equivocal cases on FISH and IHC became HER2 positive after re-testing. Time to re-testing and to definitive diagnosis on HER2 status required up to 5 weeks (either time to surgical specimen or to repeated assays on the preoperative core biopsies).

Data are shown in **Table 2.**

**Table 2:** Concordance of test results in routine diagnostic HER2 assays in breast cancer. Immunohistochemistry (IHC) and FISH between Oct 2013 and Oct 2014

	IHC score 0	IHC score 1+	IHC score 2+	IHC score 3+
FISH amplified	-	4 (1.5%)	15 (8.5%)	77 (97.4%)
FISH non-amplified	112 (98.5%)	237 (95%)	145 (83%)	1 (1.3%)
FISH equivocal	2 (2%)	7 (3%)	15 (8.5%)	1 (1.3%)
FISH monosomy	-	1 (0.5%)	-	-
N = 617	114 (100%)	249 (100%)	175 (100%)	79 (100%)

## Discussion

In our study we could demonstrate, that overall HER2 positivity rate in breast cancer increased in 2% after implementing the modified ASCO/CAP guidelines in 2013 on HER2 testing. Our data show, that this increase is mainly due to newly defined cut-offs on FISH positivity, whereas score 3+ on immunohistochemistry did not essentially influence the increase in HER2 positivity rate. Additionally, a relative high percentage of equivocal cases (up to 4%) were observed by using these guidelines. This new phenomenon in our diagnostic HER2 testing, being virtually absent prior to these guidelines, resulted in a considerable delay in the definitive pathology report on HER2 status due to re-testing.

There is only limited data on long-term impact of the modified ASCO/CAP Guidelines in the literature, as time passed since the official implementation of these guidelines encompasses around one and half years first [8–13]. Nevertheless, the limited available studies consequently report on the increase of HER2 positive breast cancer cases raining from to 1.4% to 7.3%using the 2013 ASCO/CAP guidelines [8–11, 14]. Our data of 2% increase on overall HER2 positivity corresponds to these early observations. The new guidelines offer a more wide definition on score 2+ on immunohistochemistry, which consequently may result in more equivocal cases on IHC as was reported recently [8, 10, 12]. The recommendation of the current guidelines involve reflex testing of IHC score 2+ cases [4]. As reported by Sapino et al recently on a large cohort of IHC score 2+ cases (n = 957), the percentage of amplified cases on ISH (in situ hybridisation) varies from 15–29.5%, even though the use of alternative polymerase chain reaction based technics as MLPA, the percentage of amplified IHC score 2+ cases did not exceed 25% [12]. In our experience of the last 12 months, there was no significant increase of IHC score 2+ cases (1.6% more using the 2013 guidelines), but a slight increase in score 3+ on IHC (by 1% compared to the previous years) occurred in our experience.

Consequently to increased IHC score 2+ cases, the need for reflex testing with ISH technology is also arising, resulting in higher workload in ISH, which reportedly can be up to doubling the number of ISH assays when comparing numbers from using the previous guidelines in some recent works [10, 15]. In our experience, the increase of FISH assays were mainly due to equivocal cases on ISH, as the majority of these cases were IHC score 1+ or 2+, consisting around 5% of the cohort. Following the recommendation of the current guidelines, equivocal cases on IHC and FISH need to undergo further testing either on a further tumor block or on the same sample if no further blocks are available [4]. Our cohort, we reached in 23% of the initially equivocal cases, either an amplified status in FISH or IHC score 3+. Our data on re-testing and positivity rate correspond to spare available literature data on re-testing data on IHC and/or ISH equivocal cases [9, 12].

The question of reproducibility of modified scoring criteria was addressed by Bianchi et al in a recent multicentre study [16]. Based on these data, there was a false negative rate of 24.6% between the participating institutions using the updated guidelines, mainly due to the interpretation issues, pointing out to the need of participation in internal and external quality assurance programs [16].

Although the updated guidelines address issue on analytical validity and clinical utility, there is also a recommendation on re-testing in cases with histopathological discrepancies as downgrading in histological grade on excision specimens [4, 17–19]. The question of how justified this recommendation is in the view of relative low incidence of HER2 heterogeneity and of the available resources of the given pathology institutions have been addressed recently by Rakha et al [18, 20]. Furthermore, the change in definition of IHC score 2+ subsets being scored as IHC score 1+ using the previous guidelines, raises further concern as efficacy and availabilities within the routine diagnostic service [18]. In our experience, equivocal cases on IHC and /or FISH, undergoing re-testing, resulted in a few weeks delay until a definitive diagnosis on HER2 status became possible. As also pointed out by the ASCO/CAP panel recently, guidelines need revision if further evidence based data will be available after the implementation of these recommendations [20].

## Conclusions

In conclusion it can be stated, that implementing the modified ASCO/CAP Guidelines from 2013 resulted in an increase of 2% in overall HER2 positivity rate in comparison to overall positivity rate by applying the previous ASCO/CAP guidelines. The high rate of equivocal cases (4.1%) did not considerably contribute to the increase in overall HER2 positivity, but resulted in delay of the definitive diagnosis on HER2 status. Further data and experiences from diagnostic HER2 services are needed to be collected and prospectively analysed so that adjustments in the guidelines can be met based on evidence and experience.

## Abbreviations

ASCO/CAP: American Society of Clinical Oncology / College of American Pathologists
FDA: Federal Drug Administration
HER2: human epidermal growth factor receptor 2
FISH: fluorescence labeled n situ hybridization
IHC: immunohistochemistry
